# Non-Hospitalized Long COVID Patients Exhibit Reduced Retinal Capillary Perfusion: A Prospective Cohort Study

**DOI:** 10.3390/jimaging11020062

**Published:** 2025-02-17

**Authors:** Clayton E. Lyons, Jonathan Alhalel, Anna Busza, Emily Suen, Nathan Gill, Nicole Decker, Stephen Suchy, Zachary Orban, Millenia Jimenez, Gina Perez Giraldo, Igor J. Koralnik, Manjot K. Gill

**Affiliations:** 1 Department of Ophthalmology, Feinberg School of Medicine, Northwestern University, Chicago, IL 60611, USA; 2 Department of Ophthalmology, Medical College of Wisconsin, Milwaukee, WI 53226, USA; 3 Division of Biostatistics, Department of Preventive Medicine, Feinberg School of Medicine, Northwestern University, Chicago, IL 60611, USA; nathan.gill@northwestern.edu; 4 Davee Department of Neurology, Feinberg School of Medicine, Northwestern University, Chicago, IL 60611, USAigor.koralnik@northwestern.edu (I.J.K.)

**Keywords:** optical coherence tomography angiography (OCT-A), vessel density (VD), vessel length density (VLD), long COVID, retina

## Abstract

The mechanism of post-acute sequelae of SARS-CoV-2 (PASC) is unknown. Using optical coherence tomography angiography (OCT-A), we compared retinal foveal avascular zone (FAZ), vessel density (VD), and vessel length density (VLD) in non-hospitalized Neuro-PASC patients with those in healthy controls in an effort to elucidate the mechanism underlying this debilitating condition. Neuro-PASC patients with a positive SARS-CoV-2 test and neurological symptoms lasting ≥6 weeks were included. Those with prior COVID-19 hospitalization were excluded. Subjects underwent OCT-A with segmentation of the full retinal slab into the superficial (SCP) and deep (DCP) capillary plexus. The FAZ was manually delineated on the full slab in ImageJ. An ImageJ macro was used to measure VD and VLD. OCT-A variables were analyzed using linear mixed-effects models with fixed effects for Neuro-PASC, age, and sex, and a random effect for patient to account for measurements from both eyes. The coefficient of Neuro-PASC status was used to determine statistical significance; *p*-values were adjusted using the Benjamani–Hochberg procedure. Neuro-PASC patients (*N* = 30; 60 eyes) exhibited a statistically significant (*p* = 0.005) reduction in DCP VLD compared to healthy controls (*N* = 44; 80 eyes). The sole reduction in DCP VLD in Neuro-PASC may suggest preferential involvement of the smallest blood vessels.

## 1. Introduction

COVID-19 was initially classified as an acute respiratory disease. It was soon realized that a significant number of patients exhibited persistent pulmonary, cardiac, gastrointestinal, and neurologic symptoms [1]. This phenomenon has been referred to as “long COVID”, or post-acute sequelae of COVID-19 (PASC). While the incidence of PASC is highest in those hospitalized for their severe acute respiratory syndrome coronavirus 2 (SARS-CoV-2) infection, the majority of cases are reported in individuals with mild infection [2]. According to the Long COVID Household Pulse Survey from the National Center for Health Statistics, over 17.4% of all adults in the United States have experienced long COVID, with 5.3% (14 million) currently experiencing symptoms [3]. PASC also carries a heavy economic burden, with nearly 4 million individuals in the US unable to work at any given time according to the Brookings Institute [4].

While the mechanism of PASC is not well understood, prevailing theories suggest that inflammation from SARS-CoV-2 infection triggers an immune response that affects the vasculature, as thromboembolic complications have been observed in a significant proportion of patients [5]. The retinal microvasculature may be used as a reflection of the systemic microvasculature and has revealed conflicting findings in preliminary studies of COVID-19 patients [6,7,8,9,10,11]. These studies have either focused on those recovered from COVID-19 or have failed to differentiate patients with severe infection necessitating hospitalization from those with a milder infection requiring outpatient treatment. These groups have different demographics, comorbidities, and cognitive function, suggesting that distinct mechanisms may exist [12,13].

In all of these studies, optical coherence tomography angiography (OCT-A) has served as a key tool to non-invasively provide high-resolution images of the retinal microvasculature by utilizing the movement of red blood cells as intrinsic contrast [14]. In recent years, OCT-A technology has undergone significant advancements by increasing the field of view and improving image resolution [15,16]. There are also ongoing efforts to standardize OCT-A measurements across devices, enhancing the clinical utility of this imaging modality for both ocular and systemic diseases [15,16,17,18,19].

The present study is the first to evaluate the retinal microvasculature in a cohort of non-hospitalized PASC patients exhibiting persistent neurologic symptoms (Neuro-PASC) in an effort to elucidate the pathogenesis of this debilitating condition.

## 2. Materials and Methods

This prospective observational cohort study was approved by the Institutional Review Board of Northwestern University. The study adhered to the tenets of the Declaration of Helsinki. Neuro-PASC patients were recruited from September 2021 to August 2022 and healthy controls were recruited from October 2018 to June 2022. Written informed consent was obtained from all subjects enrolled in the study.

### 2.1. Patient Criteria

Neuro-PASC patients with a positive reverse transcription-polymerase chain reaction (RT-PCR), antigen, or serology SARS-CoV-2 testing and neurological symptoms lasting ≥6-weeks from onset were included. Patients with prior COVID-19 hospitalization were excluded. Healthy controls met the following inclusion criteria: (1) absence of symptoms suggestive of SARS-CoV-2 infection during the previous months and (2) no evidence of retinal disease. Participants in either group were excluded if they (1) had a history of cataract, vitreous hemorrhage, glaucoma, high myopia, retinal occlusive diseases, choroidal atrophy, choroidal neovascularization, central serous chorioretinopathy, infectious choroiditis, fovea plana, or age-related macular degeneration; (2) had a lack of capacity to provide informed consent; (3) were unable to participate in a clinical eye examination; or (4) were younger than 18 years old. Eyes with optical coherence tomography angiography (OCT-A) scan quality (Q-score) < 6 were excluded. Sample size was determined by patient availability and willingness to participate, as this pilot study was conducted without prior data to guide a formal power calculation.

### 2.2. Evaluation Procedures

Neuro-PASC patients were examined by a board-certified ophthalmologist (MKG) and imaged by research personnel (ND). The Patient Reported Outcome Measurement Information System (PROMIS) Bank v1.0—Fatigue and PROMIS v2.0—Cognitive Function (*N* = 24) and the National Institute of Health (NIH) Toolbox v2.1 (*N* = 20) were completed at the Northwestern Medicine Neuro COVID-19 clinic. PROMIS provides a patient reported assessment of quality of life as it relates to cognition and fatigue; the NIH toolbox provides an assessment of cognitive function including processing speed, attention, executive function, and working memory [12]. These assessments have previously been described in detail by Perez Giraldo et al. [12].

### 2.3. OCT-A Imaging

The RTVue-XR Avanti system (Optovue, Inc. Fremont, CA, USA) with split-spectrum amplitude-decorrelation angiography software version (2017.1.0.151) was used to generate 3 × 3 mm (304 × 304 pixels) OCT-A images centered on the fovea [20]. The scan rate for the OCT-A device is 70,000 A scans per second with a light source at 840 nm and a bandwidth of 45 nm. This device is a modified OCT system that captures the movement of red blood cells in the retina by acquiring 2 subsequent images of the same location. Movement signals can be attributed to blood flow, which is transformed by the split-spectrum amplitude-decorrelation angiography algorithm into a three-dimensional reconstruction of the vasculature [20]. The final 3 × 3 mm scan centered on the fovea has a depth of 2 mm and contains 304 B-scans, each with 304 A-scans.

### 2.4. Image Analysis

As described previously, we used the automated AngioVue Analytics software (2017.1.0.151) for segmenting 3 vascular layers: the full retinal vascular network, the superficial (SCP) and the deep (DCP) capillary plexus [21]. The full retina slab was defined as the tissue from the inner limiting membrane to 10 μm below the outer plexiform layer [21]. The SCP was defined as the tissue from the inner limiting membrane to 10 μm above the inner plexiform layer, and the DCP was defined as the tissue from 10 μm above the inner plexiform layer to 10 μm below the outer plexiform layer [21]. Following segmentation, images were exported to ImageJ (National Institutes of Health, Bethesda, MD, USA).

We acquired repeat OCT-A scans (Neuro-PASC mean: 4.6, Controls mean: 2.0) and averaged the images to improve image quality and increase signal-to-noise ratio, as has been described previously [22,23]. Images were registered using the SCP slab via the Register Virtual Stack Slices Plugin (Feature Extraction Model  =  Rigid, Registration Model  =  Elastic) in ImageJ. The highest quality scan was used as the reference image for registration. The saved transformation matrix was applied to the full retinal slab and DCP using the Transform Virtual Stack Slices plugin. After generating the SCP, full, and DCP registered stacks, the Z-project plug-in (Projection Type = Average Intensity) was applied, and each stack was averaged based on intensity.

**Figure 1:**
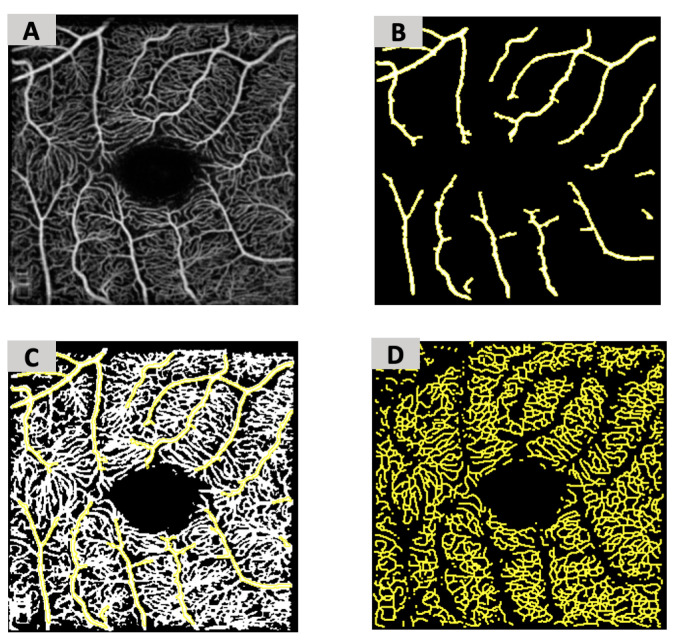
Optical coherence tomography angiography (OCT-A) image processing in Image J. (**A**) Original image of superficial capillary plexus (SCP) slab prior to processing. (**B**) Resulting large vessel mask after binarization with maximum entropy plug-in. (**C**) Binarized SCP slab used to calculate vessel density (VD) with the removed large vessels. (**D**) Final binarized and skeletonized SCP slab used to calculate vessel length density (VLD) with removal of the large vessels.

The foveal avascular zone (FAZ) was manually delineated using the full retinal slab and measured as an area (mm^2^). We binarized the large vessels using the maximum entropy plugin and saved an inverted image as a mask (Figure 1A,B). This large vessel mask was applied to the averaged images to reveal only the capillary vessels. A semi-automated ImageJ macro was then used to measure vessel density (VD) and vessel length density (VLD). We binarized the DCP and SCP using the automated global thresholding method Huang2 to convert the grayscale image into a binary white and black image that was used for quantification [24]. The white pixels represent vessel pixels, subsequently used in our VD calculation (Figure 1C). We then skeletonized the DCP and SCP to account for vessel size and the fact that the smaller central macular capillaries were below the transverse resolution of the device [21]. The white pixels here represent the length of vessels and were subsequently used in our VLD calculation (Figure 1D).

VD (mm^−1^) was defined as the area of vessel pixels divided by the total area of interest and was reported as a percentage. VLD (mm^−1^) was defined as the total length of skeletonized vessels divided by the area as previously reported [21]. The FAZ, VD, and VLD of each layer represent the primary outcome variables. The inter-rater reliability was assessed using two independent graders for the 30 (*N* = 60 eyes) Neuro-PASC patients.

### 2.5. Statistics

Inter-rater reliability was assessed using the concordance correlation coefficient. Age, sex, hypertension, and type 2 diabetes mellitus were identified as potential confounders based on their known effects on OCT-A parameters as well as the expert opinion of a board-certified ophthalmologist (MKG). OCT-A variables were analyzed using linear mixed-effects models with fixed effects for Neuro-PASC status, age, sex, hypertension, and type 2 diabetes mellitus (T2DM) and a random effect for patient to account for measurements from both eyes for some patients. Observations were weighted by the number of scans used to obtain the final OCT-A measurement. The coefficient of Neuro-PASC status was used to determine statistical significance, and *p*-values were adjusted using the Benjamani–Hochberg procedure [25].

Differences on PROMIS and NIH Toolbox Testing were determined using patient T-scores compared to the demographic-matched normative US population median of 50 using 1-sample Wilcoxon signed-rank tests [12]. Correlations between OCT-A parameters and PROMIS/NIH Toolbox measures were performed using a Spearman correlation test [12].

## 3. Results

### 3.1. Patient Demographics, Comorbidities, and Neurologic Symptoms

Thirty Neuro-PASC patients (*N* = 30, 60 eyes) and forty-four healthy controls (*N* = 44, 80 eyes) were included (Table 1). There was no significant difference in age, gender, race, and ethnicity distribution between the two groups (Table 1). Significantly (*p* < 0.001) more study subjects (*N* = 26, 86.7) were vaccinated than controls (*N* = 17, 38.6). Neuro-PASC patients were evaluated in the ophthalmology clinic on average 12.97 (range: 4–29) months from COVID-19 symptom onset.

Co-morbidities of Neuro-PASC patients are listed in Table 2 and include hypertension (*N* = 8; 26.7%), depression/anxiety (*N* = 6, 20.0%), hyperlipidemia (*N* = 5, 16.7%), and type 2 diabetes mellitus (*N* = 2, 6.7%). Following SARS-CoV-2 infection, over 80% of patients reported more than four new neurologic symptoms (*N* = 25; 83.3%) including brain fog (*N* = 29; 96.7%), anosmia (*N* = 21; 70%), dysgeusia (*N* = 20; 66.7%), headache (*N* = 20; 66.7%), and blurred vision (*N* = 17; 56.7%); complete symptomatology is shown in Table 3.

**Figure 2:**
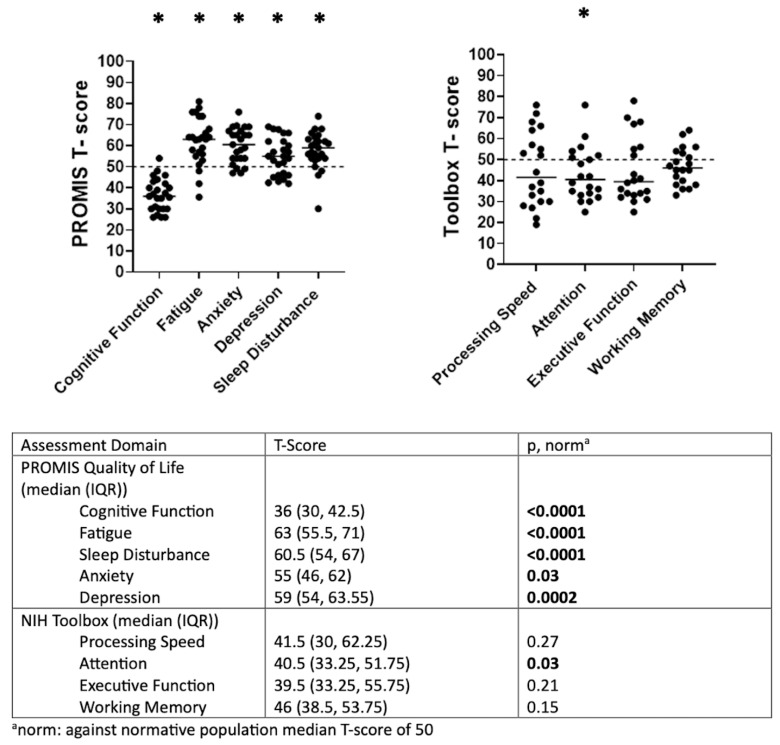
Quality of life and cognitive function results in non-hospitalized post-acute sequelae SARS-CoV-2 infection (PASC) patients with predominantly neurologic symptoms. Neuro-PASC patients exhibited a broad reduction in quality of life compared to a US normative population and significantly worse cognitive function in attention only. * *p* < 0.05.

In PROMIS testing, the Neuro-PASC cohort exhibited a statistically significant reduction in the quality-of-life domains of cognitive function (*N* = 20, 83.3%; *p* < 0.0001), fatigue (*N* = 18, 75%; *p* < 0.0001), sleep disturbance (*N* = 16, 66.7%; *p* < 0.0001), anxiety (*N* = 15, 62.5%; *p* = 0.03), and depression (*N* = 11, 45.8%; *p* = 0.0002). In NIH Toolbox testing, the Neuro-PASC cohort (*N* = 20) had a statistically significant deficit in attention (*N* = 10, 50%; *p* < 0.03). Complete PROMIS and NIH Toolbox data are shown in Figure 2.

### 3.2. Ophthalmologic Evaluation and OCT-A Parameters

Neuro-PASC patients’ best corrected visual acuity ranged from 20/20 to 20/30. Anterior segment examination, intraocular pressures, and fundus examination are reported in Supplementary Table S1. Spectral-domain optical coherence tomography findings were unremarkable.

**Figure 3:**
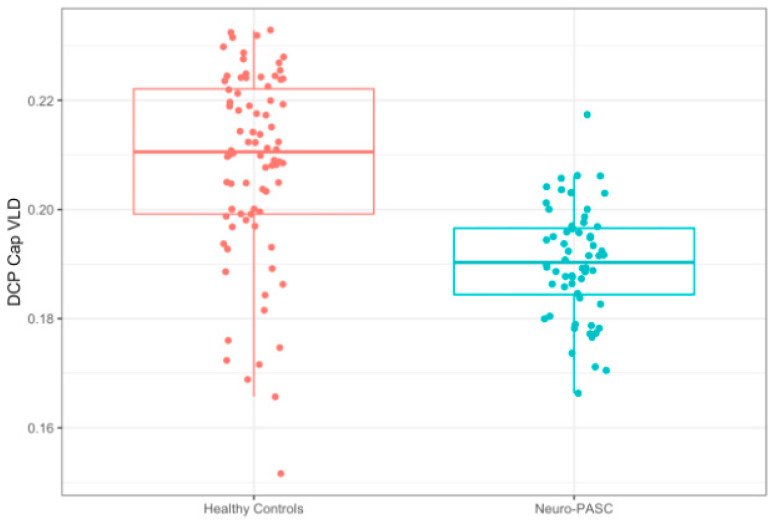
Deep capillary plexus (DCP) VLD in Neuro-PASC patients versus healthy controls. A box plot showing the difference in DCP VLD between healthy controls and Neuro-PASC patients.

The agreement between two independent scan raters (CEL and ES) was excellent with a concordance correlation coefficient of 0.999. DCP VLD was significantly decreased in Neuro-PASC patients compared to healthy controls [(ß = −0.011 mm^−^^1^, 95% CI (−0.017, −0.005 mm^−^^1^), *p* = 0.001]. FAZ [(ß = 487.507 mm^2^, 95% CI (−209.222, 1184.236 mm^2^), *p* = 0.383], SCP VD (ß = 0.018 mm^−^^1^, 95% CI (−0.007, 0.043 mm^−^^1^), *p* = 0.383), DCP VD [(ß = 0.008 mm^−^^1^, 95% CI (−0.006, 0.022 mm^−^^1^) *p* = 0.426)], and SCP VLD [ß = −0.001 mm^−^^1^, 95% CI (−0.009, 0.007 mm^−^^1^), *p* = 0.774)] were not significantly different between the cohorts. After adjustments for age, sex, hypertension, T2DM, and multiple comparisons, DCP VLD maintained a significant reduction (*p* = 0.005) in study patients. Complete results are reported in Table 4. A box plot illustrating the observed difference in DCP VLD is shown in Figure 3 and a scatter plot comparing DCP VLD with age in both cohorts is shown in Supplementary Figure S1. OCT-A parameters were correlated with PROMIS quality of life domains and NIH Toolbox cognitive test results; analysis did not reveal a significant relationship.

## 4. Discussion and Conclusions

Our study utilizes OCT-A to evaluate the retinal microvasculature in Neuro-PASC patients. We found a significant reduction in DCP VLD but not VD, suggesting a reduction in the perfusion of the smallest capillary vessels [26]. The absence of larger vessels in the DCP allows the VLD metric to reflect the ischemic status of the DCP [21]. Our results indicate that the reduction in DCP VLD in Neuro-PASC patients may reflect an ischemic state in the deep layer secondary to decreased capillary perfusion.

Prior OCT-A studies have focused on patients recovered from COVID-19 and failed to appropriately stratify patients by severity of initial infection and hospitalization status, often reporting conflicting findings. Early studies revealed a reduction in the VD of the SCP and DCP in recovered COVID-19 patients compared to controls [6,7,8,27]. These findings have not been consistent, as Savastano et al. found no differences in the SCP and DCP VD in recovered COVID-19 patients [9]. Kalaw et al. evaluated a cohort of COVID-19 convalescent patients stratified by receipt of outpatient or inpatient care as an indicator of mild or severe infection, respectively [10]. The authors showed a significant reduction in the VD of the full retinal slab and in the VD of the DCP in the severe infection group compared to the mild infection and control groups [10]. While our study also identified differential changes in the DCP and SCP vasculature, the preferential reduction in VLD over VD suggests a more selective reduction in the perfused capillary vasculature in Neuro-PASC patients. A recent meta-analysis identified reductions in vessel density in all retinal capillary plexuses of PASC patients; however, this study again failed to differentiate those with initially moderate infection from those with initially severe infection [28]. Differential reduction in the DCP relative to the SCP has been observed in early diabetic retinopathy and tends to be more severe as the retinopathy progresses, suggesting that the DCP is more sensitive to insults to the microvasculature [29]. It is possible that our cohort of Neuro-PASC patients with initially mild infection only exhibited vascular reductions in the DCP due to a more moderate level of pathology compared to those with initially severe infection who may also exhibit reductions in the SCP.

It is hypothesized that SARS-CoV-2 has the potential to cause direct damage to the central nervous system (CNS), as biomarkers of neuroaxonal damage and astrocytic activation have been shown to be elevated in acute infection [30]. The mechanism underlying this CNS damage and the associated long-term sequelae is unknown but leading hypotheses include systemic inflammation due to persistent infection and/or antigenic stimulation in PASC patients causing persistently elevated cytokine and chemokine levels [30,31]. These inflammatory mechanisms may cause endothelial dysfunction in the brain, lungs, heart, intestines, kidneys and liver [5]. Our findings of reduced capillary perfusion may support this hypothesis.

Lower retinal VLD has been reported to exhibit a significant relationship with worse cerebrovascular reactivity, reduced perfusion of the middle cerebral artery on brain MRI, and reduced cognitive function [32]. These findings suggest Neuro-PASC patients may have an underlying reduction in small vessel perfusion in the brain contributing to their observed neurologic symptoms. It is important to note that studies investigating brain MRI findings in PASC patients have largely been negative or inconclusive [33]. Vasilev et al. identified several studies that found white matter lesions—a marker of small vessel disease in the brain—in PASC patients but failed to show a correlation between these lesions and neurologic symptoms [33]. The lack of findings on brain MRI does not invalidate our findings of reduced DCP VLD in Neuro-PASC patients, as OCT-A evaluates the exquisitely small retinal microvasculature in a quantitative way that cannot be achieved by conventional brain MRI. It is possible that the reduced capillary perfusion in Neuro-PASC is too mild to be captured by MRI and can only be identified on OCT-A within the CNS, particularly in those individuals who did not require hospitalization for their initial COVID-19 infection. On the other hand, sublingual videomicroscopy has similarly shown that PASC patients exhibit a reduction in vascular density exclusively affecting small capillaries [34]. This small capillary rarefication was associated with neurocognitive symptoms, further supporting our hypothesis that the smallest vessels are preferentially affected in Neuro-PASC [34]. Flow mediated vasodilation, a non-invasive assessment of vascular function, has been used to predict both mortality and length of hospital stay in COVID-19 patients further supporting the development of vascular biomarkers—such as DCP VLD—to assist in the management and prognostication of Neuro-PASC [35].

While immune dysregulation may be common across the spectrum of PASC, unique pathophysiologic sequelae drive the different presentations of the condition. In contrast to the likely inflammatory-induced microvascular changes observed in our cohort, the gastrointestinal PASC subtype has been associated with a unique expansion in cytotoxic T cells [2]. Given that Neuro-PASC patients have been shown to significantly differ based on the severity of their initial SARS-CoV-2 infection, there is a strong case that future PASC studies should be stratified by subtype and initial infection severity. This structure advances our understanding of the pathophysiologic causes of each PASC subtype and enables the development of more targeted treatments in the future.

Our study does exhibit some limitations. First, the cohort was relatively small (*N* = 30), which limits the generalizability of the findings of this pilot study. The cohort size also resulted in the study being underpowered to identify correlations between OCT-A metrics and NIH Toolbox and PROMIS measures of cognition. Additionally, eight Neuro-PASC patients had a history of hypertension and two had a history of T2DM. These conditions are known to reduce VD and VLD while enlarging the FAZ and as such were appropriately controlled for in our study [36,37]. No effect on VD and FAZ was observed in our cohort, suggesting that these comorbidities are unlikely to be confounding our results. Reductions in VD in the SCP and DCP have been reported in those with major depressive disorder (MDD) but were not observed in our Neuro-PASC cohort [38]. Although vaccination status does significantly differ between the two groups, no correlation between COVID-19 vaccines and the retinal microvasculature has been identified [39]. Several controls were imaged prior to the onset of the COVID-19 pandemic (*N* = 17, 29 eyes), while the majority (*N* = 27, 51 eyes) were imaged during the pandemic. None of these patients reported symptoms prior to imaging suggestive of SARS-CoV-2 infection; due to testing shortages at the time of enrollment, it was impossible to test the cohort for past or present COVID-19 infection. It is possible that some controls had asymptomatic or prior infections at the time of imaging, which could introduce an unknown level of variability to the study. However, young, asymptomatic patients with a prior history of SARS-CoV-2 infection have been shown to be no different than healthy controls on OCT-A [40].

In conclusion, our study identifies retinal perfusion changes in Neuro-PASC patients with a reduction in DCP VLD, suggesting that the smallest capillary vessels are preferentially targeted. The reduction in the retinal microvasculature on OCT-A may serve as a biomarker for more diffuse small vessel disease in the brain that contributes to the cognitive dysfunction seen in Neuro-PASC patients. The correlation of OCT-A parameters with antigens, cytokines, and antibodies suspected to play a role in PASC may help elucidate the specific mechanism causing microvascular drop-out in these patients. Future studies characterizing the relationship between longitudinal changes in patient symptoms, OCT-A parameters, and plasma biomarkers of mitochondrial and metabolic dysfunction known to be altered in PASC are warranted and may establish OCT-A as an independent biomarker capable of assisting in the diagnosis, management, and prognostication of Neuro-PASC [41].

## Figures and Tables

**Table 1:** Study subjects’ demographics.

Demographics	Subjects	Controls	*p*-Value
*N*	30	44	
Age, years (mean (1SD))	52.3 (12.8)	47.5 (9.68)	0.09
Gender			0.80
Male, n (%)	9 (30.0)	12 (27.3)	
Female, n (%)	21 (70.0)	32 (72.7)	
Race, n (%)			0.67
White	20 (66.7)	29 (65.9)	
Black	2 (6.7)	2 (4.5)	
Asian	1 (3.3)	0 (0)	
Unknown	7 (23.3)	13 (29.5)	
Ethnicity, n (%)			0.21
Not Hispanic or Latino	26 (86.7)	31 (70.5)	
Hispanic or Latino	2 (6.7)	4 (9.1)	
Not Specified	2 (6.7)	9 (20.5)	
SARS-CoV-2 RT-PCR, n (%)			
Positive	25 (83.3)		
Negative	3 (10.0)		
Not Performed	2 (6.7)		
SARS-CoV-2 serology, n (%)			
Positive	10 (33.3)		
Negative	2 (6.7)		
Not Performed	18 (60.0)		
Either RT-PCR, antigen test, or serology positive, n (%)	30 (100.0)		
Vaccination Status			<0.001 *
Vaccinated	26 (86.7)	17 (38.6)	
Not Vaccinated	0 (0)	24 (54.5)	
Unknown	4 (13.3)	3 (6.8)	

* *p* < 0.05

**Table 2:** Study subjects’ comorbidities.

Pre-Existing Comorbidities	*N* (%)
Hypertension	8 (26.7)
Depression/Anxiety	6 (20.0)
Hyperlipidemia	5 (16.7)
Gastroesophageal reflux disease (GERD)	3 (10.0)
Migraines	3 (10.0)
Asthma	2 (6.7)
Type 2 Diabetes Mellitus	2 (6.7)
Gout	2 (6.7)
Hypothyroidism	2 (6.7)
Fibromyalgia	2 (6.7)
Fatty liver disease	1 (3.3)
Obstructive Sleep Apnea	1 (3.3)
Cervical spondylosis	1 (3.3)
Spina bifida	1 (3.3)
Attention Deficit Hyperactive Disorder	1 (3.3)
Bell’s Palsy	1 (3.3)
Bilateral Pulmonary Embolism/Deep Vein Thrombosis	1 (3.3)
Attention Deficit Disorder	1 (3.3)
Amyloidosis	1 (3.3)
Coronary Artery Disease	1 (3.3)
Supraventricular Tachycardia	1 (3.3)
Lymphoma	1 (3.3)
Lyme Disease	1 (3.3)
Anemia	1 (3.3)

**Table 3:** Neurologic symptoms and signs attributed to PASC.

Neurologic Manifestations/Symptoms Attributed to COVID-19	*N* (%)
≥4	25 (83.3)
Brain fog	29 (96.7)
Anosmia	21 (70.0)
Dysgeusia	20 (66.7)
Headache	20 (66.7)
Blurred vision	17 (56.7)
Myalgia	17 (56.7)
Dizziness	14 (46.7)
Pain other than chest	11 (36.7)
Tinnitus	11 (36.7)
Numbness/tingling	8 (26.7)
Other Symptoms	
Fatigue	27 (90.0)
Insomnia	17 (56.7)
Shortness of breath	17 (56.7)
Depression/Anxiety	12 (40.0)
Chest pain	9 (30.0)
Dysautonomia	7 (23.3)
Gastrointestinal symptoms	4 (13.3)

**Table 4:** OCT-A parameters and Neuro-PASC status regression coefficients, standard deviations, and *p*-values.

OCT-A Parameters	Regression Coefficient (ß) and Standard Deviation	*p*-Value	Adjusted *p*-Value
FAZ Area (mm^2^)	487.507 ± 355.474	0.17	0.383
SCP Capillary VD (mm^−1^)	0.018 ± 0.013	0.152	0.383
DCP Capillary VD (mm^−1^)	0.008 ± 0.007	0.284	0.426
SCP Capillary VLD (mm^−1^)	−0.001 ± 0.004	0.755	0.774
DCP Capillary VLD (mm^−1^)	−0.011 ± 0.003	0.001 *	0.005 *

* *p* < 0.05

## Data Availability

The data that support the significant findings of this study are included in this article. Further enquiries can be directed to the corresponding author.

## Supplementary Materials

The following supporting information can be downloaded at: https://www.mdpi.com/article/10.3390/jimaging11020062/s1, Table S1: Ocular History and Exam of Neuro-PASC patients; Figure S1: Correlation of Deep capillary plexus (DCP) VLD with age in Neuro-PASC patients versus healthy controls.

## Abbreviations

The following abbreviations are used in this manuscript:

PASC	Post-Acute Sequelae of SARS-CoV-2
Neuro-PASC	Post-Acute Sequelae of SARS-CoV-2 with Neurologic Symptoms
OCT-A	Optical Coherence Tomography Angiography
FAZ	Foveal Avascular Zone
VD	Vessel Density
VLD	Vessel Length Density
SCP	Superficial Capillary Plexus
DCP	Deep Capillary Plexus
RT-PCR	Reverse Transcription-Polymerase Chain Reaction
PROMIS	Patient Reported Outcome Measurement Information System
NIH	National Institute Health
T2DM	Type 2 Diabetes Mellitus
CNS	Central Nervous System
MDD	Major Depressive Disorder
