# Supplementary material for: Non-Hospitalized Long COVID Patients Exhibit Reduced Retinal Capillary Perfusion: A Prospective Cohort Study
Source: J Imaging. 2025 Feb 17;11(2):62. doi: 10.3390/jimaging11020062 (PMC11856302; doi:10.3390/jimaging11020062)
Supplement: Supplementary file 1 [file jimaging-11-00062-s001.zip › Supplemental Files/SupplementalTable.pdf]

| Subject ID | Duration of long-COVID symptoms at study visit (months) | Ocular Symptoms | Past Ocular History | Best Corrected Visual Acuity OD | Best Corrected Visual Acuity OS | Intraocular Pressure OD | Intraocular Pressure OS | Slit Lamp Exam | Fundus Exam | Visual Field |
|---|---|---|---|---|---|---|---|---|---|---|
| 1 | 6 | Blurry vision | None | 20/25 | 20/20 | 21 | 15 | Unremarkable | OD: pink | Full |
| 2 | 11 | Photopsia, blurry vision | None | 20/20 -3 | 20/25 +2 | 21 | 20 | OU: trace nuclear sclerotic cataracts, trace cortical spokes | OU: white without pressure temporally | Full |
| 3 | 12 | None | Myopia | 20/15 +1 | 20/20 -2 | 18 | 17 | Unremarkable | OD: few patches lattice degeneration inferiorly; OS: lattice inferotemporally | Full |
| 4 | 12 | None | None | 20/30 -2 | 20/30 -2 | 15 | 15 | OU: trace nuclear sclerotic cataracts | Unremarkable | Full |
| 5 | 4 | Blurry Vision | None | 20/20 | 20/20 | 10 | 10 | Unremarkable | OS: tiny spot of pigment superiorly | Full |
| 6 | 12 | Intermittent blurred vision, dry eye | Photopsia, blepharitis, glaucoma suspect, presbyopia | 20/20 | 20/20 | 16 | 14 | OU: trace nuclear sclerotic cataracts | Unremarkable | Full |
| 7 | 12 | Photopsia | None | 20/20 | 20/20 -1 | 15 | 15 | Unremarkable | OS: area of retinal pigment epithelium atrophy nasal to fovea | Full |
| 8 | 6 | None | None | 20/20 | 20/20 | 20 | 20 | Unremarkable | OD: slight mottling in fovea, pigmented lattice inferotemporally; OS: slight mottling in the fovea | Full |
| 9 | 20 | Reduced depth perception, difficulty tracking objects | Myopia | 20/25 -3 | 20/25 -2 | 19 | 19 | Unremarkable | Unremarkable | Full |
| 10 | 20 | Blurry vision (improves as day goes on) | None | 20/20 -1 | 20/20 -1 | 14 | 14 | Unremarkable | Unremarkable | Full |
| 11 | 12 | While sick with COVID had vision like "looking through water"; has had worsened auras with migraines including "sparkles in vision" and occasional pain behind OD | None | 20/20 | 20/20 | 20 | 15 | Unremarkable | OS: spot of pigment superotemporally | Full |
| 12 | 6 | None | Myopia | 20/20 | 20/20 | 17 | 20 | Unremarkable | Unremarkable | Full |
| 13 | 6 | None | None | 20/20 | 20/20 | 21 | 23 | OU: trace nuclear sclerotic cataracts | OD: white without pressure inferotemporally | Full |
| 14 | 10 | Subjective decrease in visual acuity both near and far | Myopia | 20/20 | 20/25 +1 | 16 | 16 | Unremarkable | OD: slight tilt of disc | Full |
| 15 | 15 | Baseline floaters, dry eyes, and flashes OS | Cataracts OU, suspected glaucoma | 20/20 | 20/20-3 | 10 | 12 | OU: trace nuclear sclerotic cataracts | OD: Druse outside superotemporal arcade OS: white without pressure temporally, epiretinal membrane | Full |
| 16 | 10 | None | Lasik | 20/25+1 | 20/20 | 10 | 10 | OU: posterior vitreous detachment, OU: trace ubclear sclerotic cataracts | OU: 1+ vascular attenuation | Full |
| 17 | 18 | None | Myopia | 20/20-1 | 20/20 | 10 | 10 | OD: trace nuclear sclerotic cataracts | OS: pigment inferotemporal periphery | Full |
| 18 | 12 | New floaters, subjective decrease in VA | None | 20/25-2 | 20/20-2 | 18 | 15 | OD: posterior vitreous detachment; OU: trace nuclear sclerotic cataracts | OD: retinal pigment epithelium atrophy superior to fovea, white without pressure inferotemporally | Full |
| 19 | 15 | Distorted vision, blurry vision, floaters | Myopia | 20/20 | 20/20-2 | 19 | 18 | OU: trace nuclear sclerotic cataracts, posterior vitreous detachment | OD: superior lattice, horseshoe tear inferotemporally with associated fluid; OU: slightly tilted disk, lattice at 12 o'clock and nasally | Full |
| 20 | 14 | None | None | 20/20 | 20/20 | 19 | 21 | Unremarkable | Unremarkable | Full |
| 21 | 20 | None | Intermittent blurred vision | 20/20 | 20/20 | 16 | 14 | OU: trace cortical spokes | Unremarkable | Full |
| 22 | 17 | None | Retinal tear OD 3/2022; Cryotherapy OU almost 40 years ago; Cataract surgery OU | 20/20 | 20/20 | 14 | 14 | OU: trace pigment, posterior vitreous detachment | OD: cryotherapy scars superiorly at 12 o'clock and inferotemporally, pigment at 1 o'clock, peripheral drusen; OS: pigment changes inferiorly, drusen superiorly; OU: trace epiretinal membrane | Full |
| 23 | 17 | None | Myopia | 20/20 | 20/20 | 13 | 14 | Unremarkable | OS: atrophic chorioretinal scar outside inferotemporal arcade | Full |
| 24 | 6 | Blurry vision | Glasses | 20/20-1 | 20/30 | N/a | N/a | Unremarkable | OS: slight mottling of macula | Full |
| 25 | 19 | Change in vision, eye pain | None | 20/20 | 20/20 | 14 | 12 | Unremarkable | Unremarkable | Full |
| 26 | 12 | Tunnel vision | Narrow angle glaucoma | 20/20-2 | 20/20-2 | 16 | 16 | OD: posterior vitreous detachment, laser peripheral iridotomy, OU: trace nuclear sclerotic cataracts | OS: peripheral superotemporal intraretinal hemorrhage | Full |
| 27 | 4 | Intermittent blurry vision | None | 20/20 | 20/20 | 18 | 17 | Unremarkable | Unremarkable | Full |
| 28 | 18 | Intermittent blurry vision OD lasting 5 minutes, intermittent temporal VFD OD | Mild myopia | 20/25-2 | 20/20-2 | 15 | 17 | Unremarkable | OU: peripheral lattice | OD hemi-field loss |
| 29 | 14 | None | Mild myopia | 20/25+1 | 20/20 -1 | 16 | 14 | Unremarkable | Unremarkable | Full |
| 30 | 29 | None | None | 20/20-2 | 20/20-1 | 15 | 15 | OU: trace nuclear sclerotic cataracts, OS: posterior vitreous detachment | OS: pigment nasal periphery | Full |
