# Supplementary figures and images for: Non-Hospitalized Long COVID Patients Exhibit Reduced Retinal Capillary Perfusion: A Prospective Cohort Study
Source: J Imaging. 2025 Feb 17;11(2):62. doi: 10.3390/jimaging11020062 (PMC11856302; doi:10.3390/jimaging11020062)

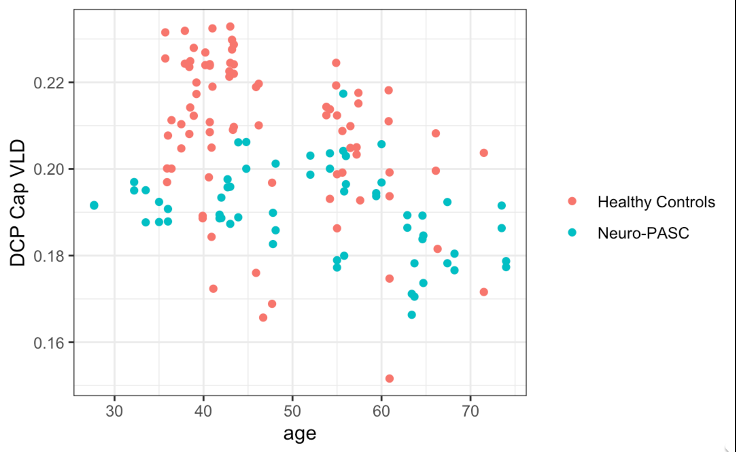

Supplement: Supplementary file 1 [file jimaging-11-00062-s001.zip › Supplemental Files/SupplementalFigure_600dpi.tif]
